# Early technical complications and long-term survival of urgent peritoneal dialysis according to break-in periods

**DOI:** 10.1371/journal.pone.0206426

**Published:** 2018-10-26

**Authors:** Kitae Kim, Young Ki Son, Su Mi Lee, Seong Eun Kim, Won Suk An

**Affiliations:** Department of Internal Medicine, Dong-A University, Busan, Korea; University of Wisconsin, UNITED STATES

## Abstract

**Background:**

Guidelines recommend a break-in period of 2 weeks before starting peritoneal dialysis (PD), but PD within 14 days is also an acceptable and safe alternative to hemodialysis (HD) in patients with an urgent need. However, the effect of the break-in period within 48 hours or later had not been evaluated for early technical complications, long-term maintenance, and survival in patients starting urgent PD.

**Methods:**

Of 360 patients with a surgically inserted PD catheter, we evaluated 190 patients who needed urgent PD and 29 patients who received conventional PD at a single center between January 2007 and December 2014 in this retrospective observational study. Enrolled patients were divided according to break-in period of <48 hours (P1) or 2–13 days (P2) before starting urgent PD. The primary endpoint was incidence of early technical complications and secondary endpoints included long-term PD maintenance, and patient survival.

**Results:**

PD was started in 103 patients (54.2%) within 48 hours and in 87 patients (45.8%) within 2 to 13 days. The incidence of early technical complication was significantly higher in P1 group (28.2%) than in P2 group (10.3%) (P = 0.002). The need for a repositioning procedure was significantly greater in P1 group (14.6%) than in P2 group (3.4%) (P = 0.009). However, we observed no significant differences between the two groups with respect to the prevalence of catheter dysfunction requiring change to HD within 6 months or incidence of peritonitis or exit-site infection. There was no significant difference in PD maintenance and patient survival according to the break-in period between P1 and P2 as well as against the control group.

**Conclusion:**

Urgent PD was associated with a low incidence of early technical complications if start was avoided within 48 hours after catheter insertion, and long-term PD maintenance was independent of the break-in period.

## Introduction

It remains unclear whether peritoneal dialysis (PD) or hemodialysis (HD) is a better modality.[1, 2] HD has been preferred for its efficacy, rapid correction of metabolic and uremic abnormalities, and convenient vascular access. However, a higher mortality rate has been reported with HD through a central venous catheter compared with PD in patients aged 65 and over who required dialysis.[3] Recently, urgent PD has been introduced as a first-line renal replacement modality if vascular access for HD is not available.[4]

Urgent PD was introduced as a feasible and safe therapeutic option. However, it is difficult to determine the safety margin of the break-in period as well as the ability of a patient to tolerate a period without dialysis. To determine the break-in period for those in need of urgent PD requires comparison of the risks and benefits of early initiation of PD. In addition, PD maintenance associated with a shorter break-in period should be considered before selecting the dialysis modality. However, there are few reports on break-in periods shorter than 7 days, and there is a lack of data on associated long-term PD survival or transition to HD.[4–9]

Accordingly, we investigated the effect of break-in periods of ≤48 hours, with an emphasis on early technical complications, long-term maintenance, and survival in patients starting PD.

## Materials and methods

### Study design and patients

This retrospective study evaluated 360 patients at a single center, Dong-A University Hospital in Korea, between January 2007 and December 2014. All patients had PD catheters implanted by experienced general surgeons using a midline or vertical incision under local anesthesia. PD catheter implantations were exclusively performed by two general surgeons and conducted by surgical method. The two surgeons had similar surgical experiences and shared the techniques. Among 360 patients, 67 patients who underwent emergent HD via femoral or jugular venous catheter placement before PD catheter insertion were excluded. These 67 patients should be treated with emergent HD and could not wait for 48 hours till PD catheter insertion.

**Fig 1:**
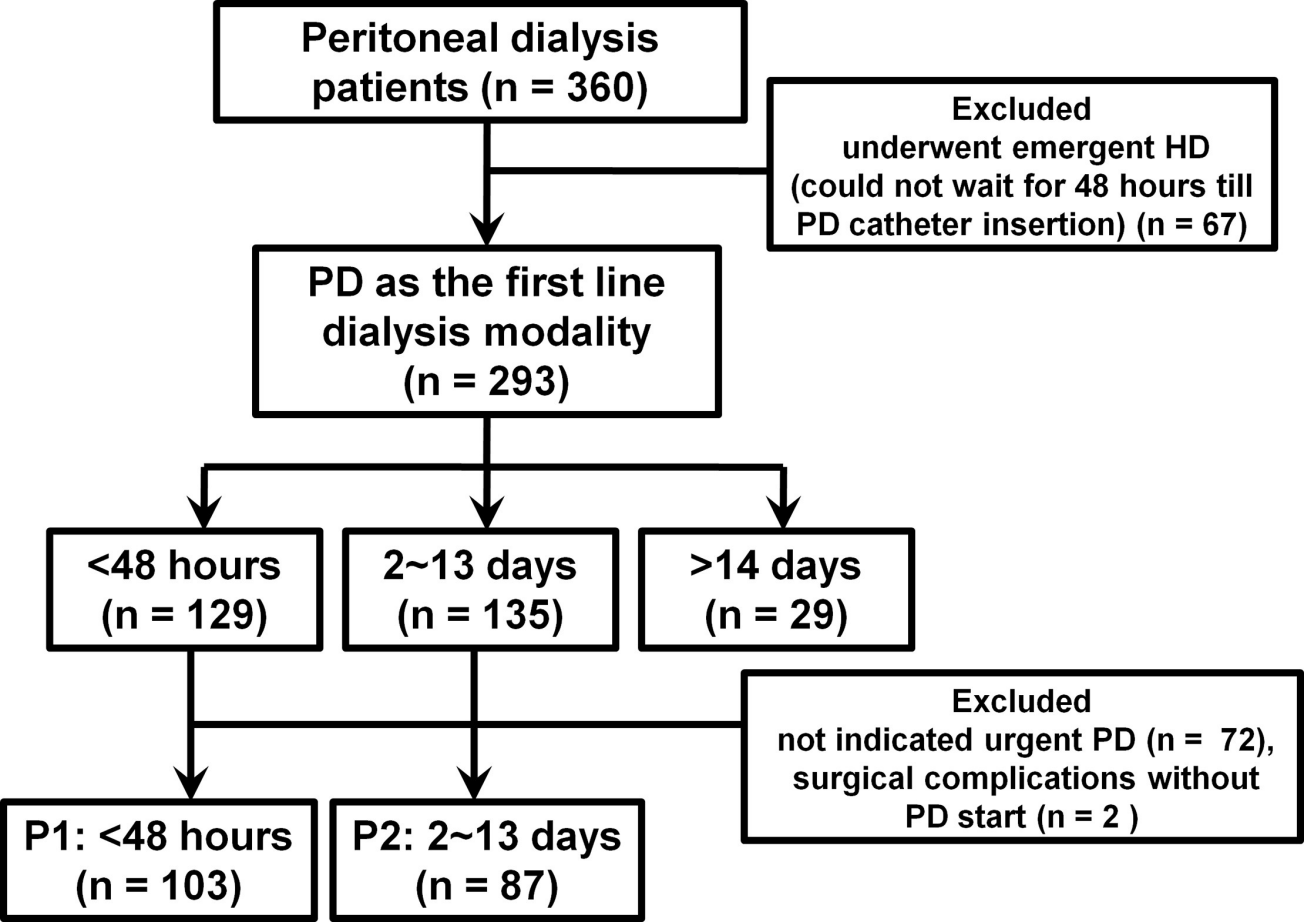
Patient flow chart.

We analyzed the data on those who started PD as a first-line dialysis modality and divided the patients into 3 groups based on the break-in period. PD was started in 129 patients within 48 hours after catheter insertion, 135 initiated PD in 2 to 13 days, and 29 started PD after 14 days or more (Fig 1). Among these, patients (n = 72) in whom urgent PD was not indicated (unmet criteria for urgent PD described below, n = 70) and experienced surgical complication related with PD catheter insertion (patients experienced bleeding, leakage and malposition without PD start, n = 2) were excluded. All of them started PD within 13 days after catheter insertion. Finally, 190 PD patients who needed urgent dialysis were included and divided into 2 groups: those who started PD within 48 hours after catheter insertion (P1 group, n = 103) and those who started within 2–13 days (P2 group, n = 87). Conventional PD was defined as the initiation of dialysis after 14 days of catheter insertion[10]. A total of 29 conventional PD patients were enrolled as controls.

This study was approved by the Dong-A University Institutional Review Board. Informed consent was waived because the study is of a retrospective design. The data including patient records and information was anonymized and de-identified prior to analysis. All clinical investigations were performed in accordance with the Declaration of Helsinki.

### Criteria and protocol for urgent PD

Indications for urgent dialysis were as follows: blood urea nitrogen (BUN) >100 mg/dL, serum creatinine >10.0 mg/dL, hyperkalemia >6.0 mmol/L in spite of medical therapy, and pulmonary congestion or uncontrolled edema.

If patients require urgent PD, the protocols of Dong-A University Hospital are as follows. Irrigation is performed 2–3 times immediately after catheter placement, using 500 mL dialysis solution. PD is started with 500 mL every 2–3 hours, 2–6 times over the following 2 days. PD volume is gradually increased to 750–1,000 mL over the next 5 days, according to the required dialysis dose. We increase the volume to 1,000–2,000 mL within 14 days, at the discretion of the physician. All patients remain supine, with minimal ambulation during the first 3 days. Initially, all patients used a lactate buffered PD solution containing 1.5% glucose concentrations (Baxter Healthcare Corporation, Deerfield, IL, USA, or Frensenius Medical Care, Bad Homburg, Germany).

### Technical complications and survival

The primary endpoint was defined as the incidence of catheter-related technical complications, such as malposition, peri-catheter leakage, omental wrapping, catheter obstruction, and the need for surgical intervention within 6 months after initiating PD. For cases who were failed conservative management to PD related complication such as sudden malposition and catheter obstruction sign, we performed laparoscopic surgical intervention and omental wrapping was diagnosed. The secondary end-point was the incidence of peritonitis, transfer to HD due to PD-related complications, late (after 6 months) catheter complications, PD survival, and overall survival.

### Statistical analysis

The data were expressed as a mean ± SD, median value, or frequency. Subject characteristics were analyzed using Student’s *t*-test for continuous variables. A Chi-squared test was used to compare categorical data between the two groups. To identify peritonitis-free survival, technical survival, and patient survival in the two groups, Kaplan-Meier analysis and log-rank tests were performed. A *P* value <0.05 was considered statistically significant, and statistical calculations were performed with SPSS software (SPSS version 18.0, Chicago, IL, USA).

## Results

### Patient characteristics

Baseline characteristics and demographic data of urgent PD groups are shown in Table 1. Mean age, sex, and prevalence of diabetes mellitus (DM) were not significantly different between the P1 group and P2 group. In addition, there were no significant differences in age, sex, and prevalence of DM between urgent and conventional PD groups. There were no significant differences in baseline laboratory data between P1 and P2 groups. However, BUN and creatinine levels were significantly higher and serum albumin level was significantly lower in the urgent PD group compared with the conventional PD group. There were no significant differences in baseline potassium, calcium, phosphorus, hemoglobin, and glycated hemoglobin levels between P1, P2, and conventional PD groups.

**Table 1:** Baseline characteristics according to break-in period in urgent PD groups.

Characteristic	Break-in Period			P value
	P1 : ≤48 hours	P2 : 2–13 days	Control ≥14 days	
**Patients, n**	103	87	29	
**Age (years)**	58.9±12.5	58.2±14.8	55.76±15.8	0.568
**Male, n (%)**	68 (66.0)	51 (58.6)	15(51.7)	0.126
**DM, n (%)**	43 (41.7)	34 (39.1)	10 (34.5)	0.478
*PD initiation time, day	0.4±0.7	5.9±2.9	20.0±8.4	<0.001
**Duration of PD, day**	980.5±685.9	715.9±575.0	1003.8±782.7	0.13
**Laboratory findings**				
Creatinine (mg/dL)	9.4±3.8	9.0±3.7	7.2±2.4	0.16
Albumin(g/dL)	3.5±0.5	3.4±0.4	3.7±0.4	0.44
BUN (mg/dL)	101.7±33.5	93.3±30.5	80.7±21.6	0.004
Potassium (mmol/L)	4.6±0.9	4.7±0.8	4.8±0.9	0.334
Hemoglobin (g/dL)	9.2±1.2	9.4±1.6	9.0±1.4	0.428
HbA1c(%)	6.9±1.5	6.8±1.4	7.5±3.0	0.338
**Indication for dialysis**				
BUN (mg/dL) >100	50 (48.5)	35 (40.2)		0.251
Creatinine (mg/dL) >10	40 (38.8)	37 (42.5)		0.605
Potassium (mmol/L) >6.0	8 (7.8)	7 (8.0)		0.943
Pulmonary edema	33 (32.0)	29 (33.3)		0.850
2 more events	26 (25.2)	19 (21.8)		0.582

DM, diabetes mellitus; PD, peritoneal dialysis; GFR, glomerular filtration rate; BUN, blood urea nitrogen; HbA1c, glycated hemoglobin

*PD initiation time : time to initiate dialysis after catheter insertion

### Early technical complications

In both P1 and P2 groups, 38 patients had catheter-related technical complications within 6 months. A higher incidence of early technical complication was noted in the P1 group compared to the P2 group (P = 0.002). However P2 group had an incidence similar to that of the conventional PD group (Table 2). In particular, malposition (22.3% vs. 4.6%) and omental wrapping (14.6% vs. 3.4%, P = 0.009), which required surgical repositioning of the of PD catheter, were noted more frequently in the P1 group compared to the P2 group. In addition, significantly more cases required surgical intervention during the overall PD period in the P1 group compared to the P2 group. However, catheter obstruction, peri-catheter leakage, and the number of transfers to HD were not significantly different. The incidence of PD peritonitis within 6 months was not different according to the break-in period.

**Table 2:** Early and long-term PD complications according to break-in period in patients who required urgent PD.

Endpoints	Break-in Period				P value
	P1 : ≤48 hours		P2 : 2–13 days	Control ≥14 days	
**Technical complications** **within 6 months, n (%)**	29 (28.2)	9 (10.3)*		3 (10.3)*	0.002
Malposition	23 (22.3)	4 (4.6)*		2 (6.9)	<0.001
Leakage	6 (5.8)	5 (5.7)		1 (3.4)	0.982
Omental wrapping	15 (14.6)	3 (3.4)*		1 (3.4)	0.009
Obstruction	1 (1.0)	1 (1.1)		0	0.904
Transfer to HD	2 (1.9)	3 (3.4)		0	0.518
Surgical intervention	15 (14.6)	3 (3.4)*		1 (3.4)	0.009
**Peritonitis within 6 months, n (%)**	10 (9.7)	9 (10.3)		5 (17.2)	0.884
**Technical complications, n (%)**	34 (33.0)	14 (16.1)*		4 (13.8)*	0.007
**Surgical intervention, n (%)**	18 (17.5)	3 (3.4)*		1 (3.4)	0.002
**Causes for drop-out, n (%)**	59 (57.3)	42 (48.3)		11 (37.9)	0.215
Death	30 (29.1)	23 (26.4)		5 (17.2)	0.680
Kidney transplantation	9 (8.7)	4 (4.6)		1 (3.4)	0.260
Transfer to HD after 6 months	18 (17.5)	11 (12.6)		5 (17.2)	0.356
Event (death or HD), n (%)	50 (48.5)	38 (43.7)		10 (34.5)	0.503

PD, peritoneal dialysis; HD, hemodialysis

*P < 0.05, compared to ≤48 hours group

### Long-term complications and PD survival

**Fig 2:**
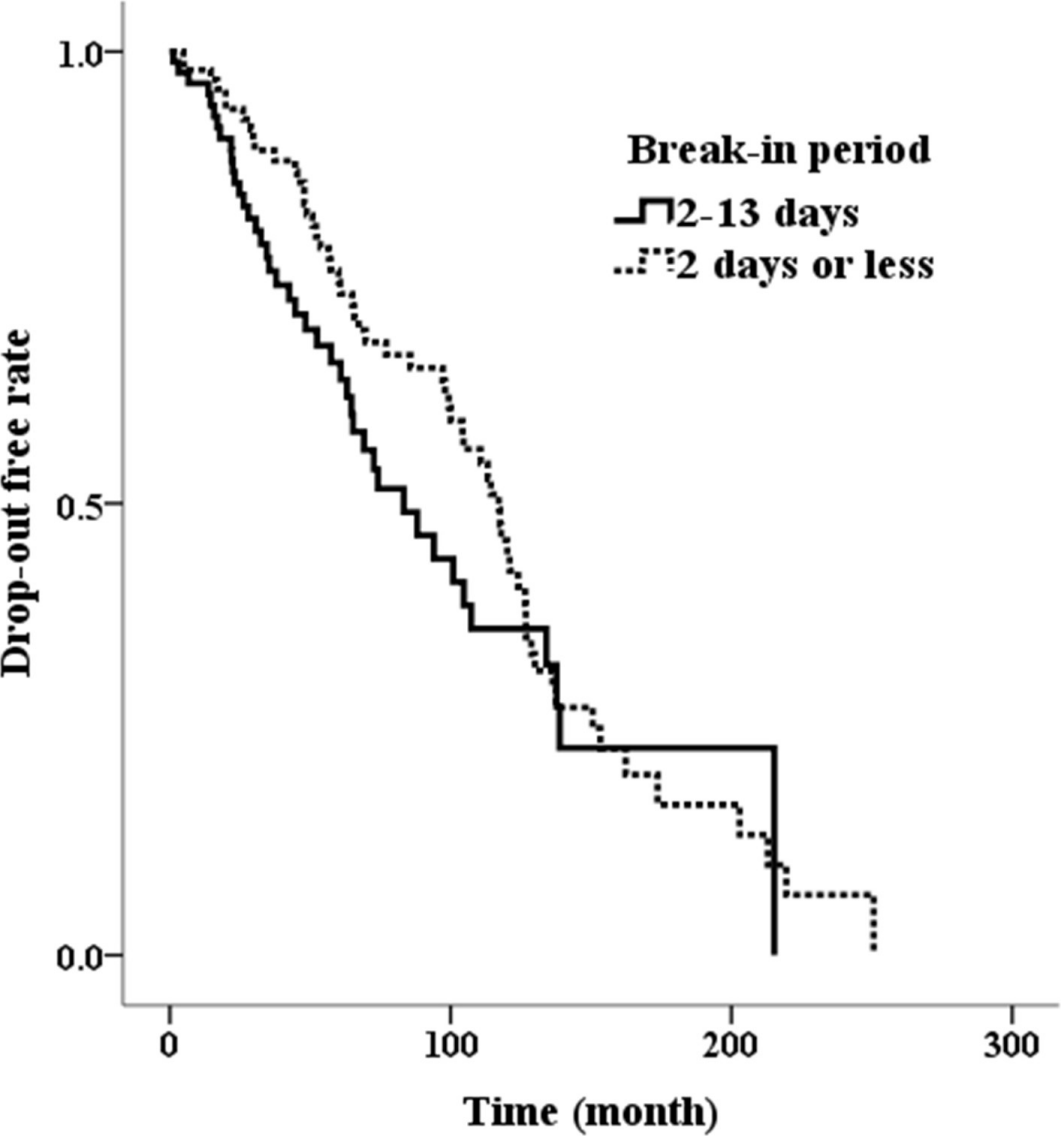
Comparison of drop-out free rate between the group that initiating break-in within 48 hours and 2~13 days (log-rank, *P* = 0.205).

The incidence of technical complication over the entire duration of PD in the P1 group was also greater than in the P2 group. However, there were no differences in drop-out rate due to transfer to HD, kidney transplantation, or death among the three groups. Overall cumulative PD survival showed similar Kaplan-Meier curves between P1 and P2 groups (log-rank, *P* = 0.205) (Fig 2). There were no differences in cumulative PD survival between the urgent PD groups and the conventional PD group (log-rank, *P* = 0.073).

## Discussions

This retrospective study found that urgent PD initiated within 2 to 13 days, i.e., with an average 6-day break-in period, was safe without serious complications. In addition, early technical complications and long-term PD survival, including PD maintenance and patient survival, were not different between urgent PD groups and the conventional PD group with a 2-week break-in period. However, the urgent PD group with ≤ 48 hours break-in period had a higher risk of early technical complications such as malposition and omental wrapping compared to the group with a 2 to 13 days break-in period. Acutely started PD within 24 hours showed a higher rate of mechanical complications compared to that in planned start PD with break-in periods >12 days in a previous retrospective study.[11] Therefore, we suggest that urgent PD can be relatively safe, with reduced incidence of early technical complications, when initiated 48 hours after PD catheter insertion. Our data suggested that a minimum 48-hour break-in period might be a milestone in the prevention of early technical complications. However, physicians should monitor patients to determine the ability to tolerate 48 hours without PD after catheter insertion. It is much safer to wait 6 days after catheter insertion to start low-volume (500 ml) PD solution and to allow enough time to achieve full volumes based on our study. Our methods were similar to those using automated PD or continuous ambulatory PD (CAPD) following acute renal failure, and cannot only reduce the occurrence of technical complications by using small volumes of dialysate, but also effectively remove overhydrated fluid and small solutes and electrolytes, especially potassium, through a short dwell time and rapid cycling within the first 24 hours.[11, 12]

Emergent PD defined as dialysis therapy could not be delayed for 48 hours. Therefore, PD catheter was inserted and PD was started within 48 hours in emergent cases. Urgent PD defined as requiring dialysis in less than 2 weeks but able to delay PD catheter insertion for more than 48 hours, with medical treatment alone. [4] Several guidelines recommend waiting at least 14 days after implantation of a PD catheter, if possible, to prevent bleeding or catheter leakage.[10, 13] However, it is difficult to wait 14 days, because treatment with medical therapy alone may be harmful to patients. In these situations, we recommend starting urgent PD without waiting 14 days, and deferring consideration of HD catheter insertion. Urgent PD is now an accepted modality, especially in patients who are appropriate candidates for PD as renal replacement therapy.[4, 14] However, physicians should select central venous catheter insertion for emergent HD in patients who cannot wait 48 hours till PD catheter insertion. If patients can wait at least 4 days (2 days waiting for PD catheter insertion plus 2 days break-in periods for urgent PD start, to reduce early technical complications), urgent PD is safe, and can achieve long-term PD and patient survival, based on our study. From these encouraging results, PD can be useful in cases needing urgent dialysis therapy.

Among early complications, malposition of the PD catheter, with the catheter tip located outside of the true pelvis on a simple abdominal X-ray, can worsen with immobilization due to surgical site pain or decreased bowel motility and constipation. When patients start urgent PD within 48 hours after catheter insertion, using a laxative to initiate bowel motility may be one option for prevention of malposition. Use of a laxative and ambulation may be helpful for repositioning of the catheter if malposition has occurred.[15] If conservative measures fail, laparoscopic repositioning or omentectomy should be performed.[12, 15–17] In spite of an increase in early technical complications, the P1 group also showed a longer duration of PD maintenance compared to the P2 group. This result might be influenced by proper timing of surgical intervention. A previous study suggested that laparoscopic internal fixation was associated with reduced catheter migration as well as maintenance of PD.[16] We performed laparoscopic internal fixation and omentectomy for correction of malposition caused by omental wrapping in this study. Although a previous study reported that the risks of malposition or omental wrapping after PD catheter insertion were associated with DM, younger age, serum albumin and use of a straight catheter, our data showed no significant differences.[18, 19] However, PD start within 5 days after PD catheter insertion and a previous history of abdominal surgery were associated with technical complications such as malposition or omental wrapping. These reports were in keeping with our findings showing an association between shorter break-in time and malposition. Simultaneous preventive laparoscopic PD catheter insertion and internal fixation may be considered if patients have a high likelihood of needing PD within 48 hours after catheter insertion.

Leakage is a major concern when starting PD urgently without a 2-4-week healing period.[20, 21] Ghaffari *et al*. reported higher frequency of leakage in urgent-start PD group than in non-urgent-start PD group.[4] However, most leaks were minor and managed with less effort except for 2 cases (11.1%).[4] Early dialysate leakage is defined as occurring within 30 days after insertion, and is usually associated with catheter implantation at the exit or incision site. Higher risk for dialysate leakage was reported with a midline incision compared with a paramedian incision, as well as with dialysate volume >500 ml and PD initiation within 10 to 14 days after implantation.[20] To minimize the occurrence of peritoneal leaks, Jo *et al*. reported use of a modified percutaneous catheter implantation technique, as in CAPD, and started dialysis immediately after catheter insertion with 500 ml of low-volume dialysate every 3 hours for the first 3 days.[9] They reported peritoneal leakage in only one case. Yang et al. also reported use of low-volume dialysate as described above, in an early-start PD group, with initiation of dialysis within 14 days after catheter insertion, with dwell volume of 750 ml and more than 4 cycles in a day, depending on uremic symptoms.[7] The dwell volume was gradually increased to 1,500 ml at 12 days after break-in, if no complications were noted during the incremental process. They compared the results in a late group that initiated dialysis 12 days after catheter insertion, and found no association between shorter break-in time and peritoneal leaks. Our study showed a similar incidence of leakage according to the break-in period in urgent PD groups using low initial dwell volume. Therefore, leakage may not be a major concern in urgent PD if patients can be managed using low initial dwell volume.

This study had some limitations. First, the design of this study was retrospective. Second, the number of control group with break-in period as guideline recommended is too small.

## Conclusions

In conclusion, initiating urgent PD within 2 to 13 days after catheter insertion may prevent early technical complications such as obstruction and malpositioning requiring surgical intervention, compared to the outcomes with a ≤48-hour break-in period. In addition, compared to conventional PD, urgent PD showed similar long-term maintenance, regardless of the break-in period.

## Supporting information

S1 Dataset This is data of included patients (n = 219). (XLSX)
